# Supplementary material for: Demographic and behavioural drivers of intra-urban mobility of migrant street children and youth in Kampala, Uganda
Source: PLoS One. 2021 Feb 18;16(2):e0247156. doi: 10.1371/journal.pone.0247156 (PMC7891785; doi:10.1371/journal.pone.0247156)
Supplement: Attachment — Submitted filename: Response to Reviewers Comments PlosONE Jan 24.2021_.docx [file pone.0247156.s002.docx]

**Authors Response to Reviewer's Comments**

**Comments to the Author**

1. If the authors have adequately addressed your comments raised in a previous round of review and you feel that this manuscript is now acceptable for publication, you may indicate that here to bypass the “Comments to the Author” section, enter your conflict of interest statement in the “Confidential to Editor” section, and submit your "Accept" recommendation.

Reviewer #1: (No Response)

Reviewer #2: All comments have been addressed

Reviewer #3: (No Response)

Response: Done

Response: We believe that all comments have been addressed unless you have any further concerns on the negative binomial regression model used.

2. Is the manuscript technically sound, and do the data support the conclusions?

The manuscript must describe a technically sound piece of scientific research with data that supports the conclusions. Experiments must have been conducted rigorously, with appropriate controls, replication, and sample sizes. The conclusions must be drawn appropriately based on the data presented.

Reviewer #1: No

Reviewer #2: Yes

Reviewer #3: No

Response: Our data supports the study findings and conclusions, using the negative binomial regression as suggested by Reviewer 3. We consider that this model is appropriate to yield valid findings.

3. Has the statistical analysis been performed appropriately and rigorously?

Reviewer #1: No

Reviewer #2: Yes

Reviewer #3: No

Response: Our data supports the study findings and conclusions, using the negative binomial regression as suggested by Reviewer 3. We consider that this model is appropriate to yield valid findings.

4. Have the authors made all data underlying the findings in their manuscript fully available?

The [PLOS Data policy](http://www.plosone.org/static/policies.action#sharing) requires authors to make all data underlying the findings described in their manuscript fully available without restriction, with rare exception (please refer to the Data Availability Statement in the manuscript PDF file). The data should be provided as part of the manuscript or its supporting information, or deposited to a public repository. For example, in addition to summary statistics, the data points behind means, medians and variance measures should be available. If there are restrictions on publicly sharing data—e.g. participant privacy or use of data from a third party—those must be specified.

Reviewer #1: Yes

Reviewer #2: Yes

Reviewer #3: Yes

5. Is the manuscript presented in an intelligible fashion and written in standard English?

PLOS ONE does not copyedit accepted manuscripts, so the language in submitted articles must be clear, correct, and unambiguous. Any typographical or grammatical errors should be corrected at revision, so please note any specific errors here.

Reviewer #1: Yes

Reviewer #2: Yes

Reviewer #3: Yes

6. Review Comments to the Author

Please use the space provided to explain your answers to the questions above. You may also include additional comments for the author, including concerns about dual publication, research ethics, or publication ethics. (Please upload your review as an attachment if it exceeds 20,000 characters).

**Response to the above 5 questions:** The authors repeated the analysis using the negative binomial regression as advised by the third Reviewer. We note that Reviewers 1 and 2 had earlier okayed Poisson regression. This implies that the comments by the first and third reviewers somewhat contradict. In this regard, authors have considered the negative binomial regression as the final model as it best suits the study outcome count variable.

Point by Point Response to Reviewers comments

**Reviewer #1:** It is evident that this paper has been revised to address reviewers’ comments, and the authors are commended for taking note of some of the points raised. That said, I still find it hard to follow some points and the logic of the analysis undertaken. My main suggestion to authors is to consider abandoning the intra-urban mobility as outcome variable. This outcome variable is of particular concern since not all street youth answered the question on number of times moved.

Response. As earlier explained in the previous response to your initial comments, intra-city mobility of street children and youth has been a major concern by the city authorities for decades, to an extent that the city authorities have enacted ordinances that prohibit the public from supporting street children with food or money since they do not want them on the city streets. This is a human right violation.

For decades, government’s efforts to chase away street children from the streets including repatriation to their rural districts has been futile. The push factors are well documented including the biting poverty in the rural areas of Uganda which forces the young people to move to urban cities mainly in search for economic opportunities. In this study, we only focus on rural-migrant street children as the primary focus of our research for whom little is known.

The choice of our outcome variable is relevant to the field of migration and can only be considered important by scholars in the field of migration and cities and the Uganda government and urban authorities. Migration and mobility are known social determinants of health and should not be overlooked in health research. Intra urban Migration/mobility are realities that the urban health system must deal with if universal access to health care is to be achieved without leaving anyone behind.

Instead, this data may be especially useful in looking at whether in-migrant street youth are involved in sex work, or even use health services, more/less than non-migrant street youth. Indeed, this is related to the policy implications mentioned in conclusion. I still feel at a loss in following the logic of the paper. For example, what are the mechanisms that lead involvement in sex for money to higher mobility? Or why would higher income lead to greater intra-urban mobility? Below I raise my concerns (in no specific order).

**Response.** The context of sex work in Kampala city is quite complex and unique. In one of the papers we are writing, our study indicates that street children and youth engage in this risky behaviour as a survival mechanism and not out of their own wish. Street children is being sexually exploited. Sex work spots in Kampala are often located along the streets, bars and hotels and cassinos. Street children will tend to move and stay closer to hot spots to target workers for money. Sex workers by nature are seasonal migrants – move from place to place in search for clients. Understanding of this local context is critical to appreciating our study findings. Sex work is still not legal in Uganda and its association with intra-urban mobility should raise concern to the Ugandan government and city authorities and should guide policy reforms and implementation including regulating sex work and combating sex exploitation of street children and youth in Kampala and other cities.

1. If you hypothesis that migrant street youth are likely to experience disproportionate intra-urban mobility compared to native Kampala dwellers, then this should be examined. It seems though that the questionnaire wasn’t built to allow for such analysis. Therefore, it is important to back this assumption of higher intra-urban mobility among in-migrants with substantial references.

Response. Indeed, as you rightly put it, our questionnaire focused on street children and youth with a rural-urban migration experience. In that regard, our study cannot compare migrants with non-migrants but only does so by duration of stay in the city among migrant street children and youth, using a cut off of 2 years which similar studies have used to categorise migrants as short or long term. So, our hypothesis speaks to the duration of stay of the migrant street youth (and not non-migrants) who are the focus of the study.

2. Of course since street youth are examined a clear definition of residence is not possible- and neither is it easy to look at physical boundaries of their movement. Yet, this is important. If a person moved from say the bus stop on road A to a temporary shelter on Road A, less than 50m away- the meaning of this move is different to moving say to a market on Road B 2km away (in a different parish). In the first move, this person remains in the same area- meets the same people most likely- know where to get food there etc. In the second move, the person may not have a social network, may be more vulnerable etc. Therefore, the moves are essentially different and shouldn’t be combined. Thus, I find that the outcome variable has little meaning.

Response. Our paper sought to examine intra-city mobility and not residential mobility since it is not practical within our context. Street children and youth in Kampala stay in dilapidated temporary shelters within city spaces, some stay in shacks in open markets/places and shop verandas while other have found the street as their permanent home. These places and streets addresses are not demarcated. We therefore focus on intra-city movements of the street children which is the main concern of the city authorities as it increases them to vulnerabilities to poor health and risky behaviours. It is also not possible to measure distances moved. In the first place, the streets are not properly demarcated and street children do not even know specific names of places they stay. You are asking for data that is not practically available.

As earlier described in the paper, Kampala City does not have any demarcations of neighbourhoods and movements by street children are often short – along the same street, cyclic and without a clear pattern. While our research attempted to geo-map the different areas/street locations in which street children and youth live or congregate during daytime, both the first and second reviewers observed that the maps did not add value to the findings, and we had to drop them at the second iteration. We do not think it would be desirable to revert to the same now.

3. On page 8, you indicate that in the Poisson model you only include covariates that were significant in bivariate analysis. As far as I understand this is misleading. A covariate may be theoretically important in explaining intra-urban mobility and should therefore remain in the model even if not statistically significant. Moreover, there may be a covariate that is not significant in bivariate analysis but is important to control for the other effects of the covariates in the model.

Response: We have applied a mixed selection using Bayesian criteria and added additional known predictors or cofounders into the final negative binomial regression, irrespective of the 5% significance threshold at bivariate analysis. We hope this is acceptable to you.

4. Although the model indicates statistical relationship between covariates and intra-urban mobility, this relationship is not necessarily causal, and I would refrain from using the term “drivers”. If the authors are specifically interested in intra-urban mobility this still could be used to explain use of health services for example (rather than the other way round). Authors should consider reverse causality.

Response. Our very initial paper did not have drivers. It is at the recommendation of the first (yourself) and second reviewer that we adopted drivers in the title, which we think is still reasonable. In that regard, we have maintained the title as is. We highlight the issue of causality as a limitation of the cross sectional study.

Overall, I believe the authors have important data on a topic that is understudied and combining the data with qualitative analysis provides a richer perspective. However, how the data is used needs to be reconsidered.

Response. Unfortunately, we do not have any other data apart from what is presented in the paper. We conducted a few qualitive interviews to further elucidate aspects of intra-urban mobility that could not be easily explained by the questionnaire. Our understanding is that we have presented both qualitative and quantitative data in a corroborative and logical way. Our topic is novel and is the first study of its kind to interrogate intra-urban mobility of street children and youth. It formas a starting point for future work on intra-city migration.

**Reviewer #2:** The authors have adequately addressed the comments that I raised. I found the manuscript generally well written.

Response: So, noted, thank you.

**Reviewer #3:** Beside reading this paper, I have also read the two reviews of the original paper, the decision letter of the editor, and the responses of the authors to the comments by the editor and the reviewers.

This paper falls within the broad theme of wellbeing of migrant children and youth in large cities in developing countries. Their migration is often driven by poverty, joblessness and desperation in rural areas. They migrate to the city to escape the dire conditions in the home region and, to the extent that they can earn income in the city in the informal economy and perhaps even remit some money back to the family in the rural area, such migration can be welfare enhancing.

However, many of these children and youth end up in situations that violate the 1989 United Nations Convention of the Rights of the Child (UNCORC). They have no access to adequate housing, education and health care; and they are often exploited economically and sexually. Hence, research that informs on the characteristics of these children and the problems they face is important for designing policies that can protect them and enhance their wellbeing.

This paper provides a case study of this issue by studying migrant street children and youth in Kampala. The focus is on their intra-urban mobility. As the title suggests, the paper is concerned with the correlation between intra-urban mobility and the socio-demographic characteristics of these young migrants. The paper also investigates the correlates between mobility and some aspects of their sexual behaviour (by the way, it is safe to assume that the survey responses that close 90 % or more are not involved in sex work is an underestimate of engagement in sex work, particularly when their responses are censored or influenced by their “street uncles”).

However, it isn’t clear at all why we should care about the intra-urban mobility of migrant street children. On the one hand, the qualitative research suggests that their intra-urban mobility is due to “push factors” such as a lack of safety and inadequate shelter. On the other hand, intra-urban mobility may be a voluntary response by the child or youth to reap greater earnings opportunities at a different location. The paper argues on pp. 17-18 that the findings have implications for policy, but it is entirely unclear what these implications are.

Response. As earlier explained in the paper and previous response to the comments made by the initial reviewers, the topic of intra-city mobility of street children and youth remain relevant to the city authorities. The push factors are well documented elsewhere, and we found it not necessary to focus on them. Our focus on studing intra-urban mobility and not rural-urban migration. In this study, we only focus on rural-migrant street children as the primary focus of our research for whom little is known. The choice of our outcome variable is relevant to the field of migration and cities and scholars of migration health studies.

To the best of our knowledge the policy implications highlighted in the discussion are straight forward and relevant to government and Kampala Capital city Authority in addressing street children and youth’s intra-city mobility and its associated challenges, especially sex work and sexual exploitation. They also suit the local policy and environmental contexts of the study. We highlight recommendations including regulation of sex work in the wake of sex exploitation, which is a big concern in Uganda. Establishing of a longitudinal database for city residents is a strong recommendation and could help address some of the issues which our study was unable to provide - explore the drivers of intraurban mobility to the fullest.

If mobility is the response to negative push factors, the local government should aim to provide better shelter and reduce the level of street crime. This would then lower the intra-urban mobility of street children and that would be a good thing. However, if intra-urban mobility is the means by which the children can gain a greater income, mobility should be encouraged. The statistical analysis in this paper does not help us to understand what the impact of intra-urban mobility is on the children’s wellbeing.

Response: We would like to state that the impact of intra-urban mobility on children wellbeing is a novel topic but is not the main study question for our research. We set out to investigate the drivers of intra-urban mobility of migrant street children and youth. Perhaps future research could attempt to explore that. We have acknowledged the safety issue of street children and youth in the second last paragraph of the background. Crime was not a major finding of the study, although, we found it important to include in the discussion.

If the objective of this paper is simply defined as an inquiry into the characteristics of migrant street children and youth, then that can also be a perfectly legitimate research objective (but of lesser importance), provided the analysis is done well and is convincing. However, while this paper has some strong aspects, the analysis is technically flawed.

Response: The importance of this study has been well articulated in the background and the discussion. The analysis was re-done using the negative binomial regression as you suggested.

The strong aspects of the research reported in this paper are the sampling strategy and the use of mixed methods (analysis of qualitative and quantitative data).

Nonetheless, I concur with reviewer #1 that the statistical analysis is flawed. Let me elaborate. If we define the variable of interest as the number of intra-urban moves that the child or young person makes after arriving in Kampala, this outcomes variable is an integer that takes on the values 0, 1, 2, …. The Poisson regression model can be an appropriate model for quantifying the determinants of this mobility process. At the top of page 8 the authors argue that the Poisson regression assumptions have been satisfied. However, rather than testing for the negative binomial model (and hence overdispersion) as an alternative, they simply note that there is “minimal data dispersion”. But in a Poisson process the mean and the variance are the same, whereas in the Kampala data the variance is almost twice the mean!

Response: We have repeated the analysis using negative binomial regression which best suits our data and taken into consideration other potential predictors while controlling for confounding. However, both Poisson and negative binomial regression models yielded similar findings.

Even more concerning is, as reviewer #1 notes, that no account is taken of varying exposure across sampled individuals. The Poisson process has the property that if the expected number of events over a period of length T is E, the expected number of events over a period of length 2T is 2E. In other words, the number of months between the interview and the year and month in which the person entered Kampala needs to be taken into account. This information was collected (see Table 1) and should be inserted in the exposure option in the Stata command poisson.

Response: Our data is non-longitudinal and therefore not feasible to account for the time exposure aspects as you suggested. We have re-analysed the data using the three models: Poisson, negative binomial regression and OLS model as suggested by all the reviewers. We present the results from all the two regression models in Table 2. In this regard, the OLS is not our preferred model based since it does not predict the outcome perfectly well compared to the negative binomial regression. However, the negative binomial regression best fits our data and therefore the most appropriate.

An alternative approach is to calculate the number of moves per month of residence in the city and to run an OLS regression model with this statistic as the dependent variable. For this relatively large sample of 412 observations, this OLS regression is probably not a bad approximation to identifying the statistically significant determinants of intra-urban mobility. The distinction made by the authors between duration of stay > 2 years and duration of stay < 2 years is too coarse.

Response: We have maintained the negative binomial regression as the most appropriate model that fits our data. While the OLS is possible alternative, unfortunately, it does not predict our outcome perfectly. Only one variable (gender) was statistically significant. Thus, we present findings from all three models as proposed by all the three reviewers. Our data is non-longitudinal and does not capture the moves per month of residence over the duration of stay of 15 years. Previous studies in Uganda have used the 2-year cut off for migration status and that is plausible within the context of internal migration in Uganda (see reference).

The numbers of observations that were used in each of the bivariate regressions and in the multivariate regression in Table 2 were actually not stated but in many cases the number was less than 412 due to missing data. I think that this problem could have been overcome by using some form of data imputation, which can be done manually or by means of Stata.

Response: An explanation for the missing values is provided in the data analysis section of the manuscript.

Another important issue in this context is the assumption of behavioural homogeneity. It is very likely that the Poisson model differs structurally across gender or age (12-17 versus 18-24). This can be tested by running separate regressions for the sub-samples. It is noted on p.8 that further stratification by age is not possible, due to missing data, but imputation may ameliorate this. Ditto for regressions by gender.

Response: Sub-analysis by age and gender did not yield any significant differences and as such, we think this is not worth reporting in the findings.
Table 2: The number of observations in each of the bivariate regressions and in the multivariate regression should be stated in the Table. Additionally, there are no robustness checks of the multivariate model. For example, log likelihood tests could be used to check the importance of the sexual behaviour-related variables.

Response: Table w has been updated to include multivariate findings from the three analysis models.

Also, there does not seem to be a full correspondence between the descriptivism in Table 1 and the regressors in Table 2. For example, why is “highest education attained” not in Table 1?

Response: We have included highest education variable in table 2.

**Minor points**

Abstract: “continuous scale” should be “integer scale”

Abstract: just stating IRR=0.67 for gender does not inform the reader that the mobility is less among girls.

Response: Due to the limitation on word limit, we present the findings with no detailed explanations, which we think is fine for the readers to appreciate the key study findings at a glance.

Abstract: “causal” should be “casual” By the way, the findings with respect to “Personal safety” and “cost of place of stay” are not shown in the regressions in Table 2.

Response: These are qualitative findings which are derived from data presented in the results section and therefore are not part of the quantitative analysis. These data are important to be included in the abstract.

p.4, second para.: because the data come from a larger cross-sectional study, there should be a reference to a report or article that describes this larger study, as well as acknowledgement of the funding for this larger study.

Response: The main paper is still in press and therefore cannot be referenced at the moment.

Table 1: In-school, all respondents: the frequency is 35, not 55.

Response: The correct figures are n=337 for out of school vs n=55 for in-school. This is a valid finding.

p.16, 4th line from bottom: if intra-urban mobility may not be predicted by duration of stay, the stochastic process is not Poisson!

Response: This has been addressed in the analysis.

7. PLOS authors have the option to publish the peer review history of their article ([what does this mean?](https://journals.plos.org/plosone/s/editorial-and-peer-review-process#loc-peer-review-history)). If published, this will include your full peer review and any attached files.


If you choose “no”, your identity will remain anonymous but your review may still be made public.


**Do you want your identity to be public for this peer review?** For information about this choice, including consent withdrawal, please see our [Privacy Policy](https://www.plos.org/privacy-policy).

Reviewer #1: No

Reviewer #2: No

Reviewer #3: No
